# The Dose Optimization and Evaluation of Image Quality in the Adult Brain Protocols of Multi-Slice Computed Tomography: A Phantom Study

**DOI:** 10.3390/jimaging9120264

**Published:** 2023-11-28

**Authors:** Thawatchai Prabsattroo, Kanokpat Wachirasirikul, Prasit Tansangworn, Puengjai Punikhom, Waraporn Sudchai

**Affiliations:** 1 Department of Radiology, Faculty of Medicine, Khon Kaen University, Khon Kaen 40002, Thailand; kanokpat.w@kkumail.com (K.W.); prasit_ta@kkumail.com (P.T.); pungjai4327@hotmail.com (P.P.); 2 Nuclear Technology Service Center, Thailand Institute of Nuclear Technology, Nakhon Nayok 26120, Thailand; newsudchai18@gmail.com

**Keywords:** dose optimization, iterative reconstruction, image quality, radiation dose

## Abstract

Computed tomography examinations have caused high radiation doses for patients, especially for CT scans of the brain. This study aimed to optimize the radiation dose and image quality in adult brain CT protocols. Images were acquired using a Catphan 700 phantom. Radiation doses were recorded as CTDI_vol_ and dose length product (DLP). CT brain protocols were optimized by varying parameters such as kVp, mAs, signal-to-noise ratio (SNR) level, and Clearview iterative reconstruction (IR). The image quality was also evaluated using AutoQA Plus v.1.8.7.0 software. CT number accuracy and linearity had a robust positive correlation with the linear attenuation coefficient (µ) and showed more inaccurate CT numbers when using 80 kVp. The modulation transfer function (MTF) showed a higher value in 100 and 120 kVp protocols (*p* < 0.001), while high-contrast spatial resolution showed a higher value in 80 and 100 kVp protocols (*p* < 0.001). Low-contrast detectability and the contrast-to-noise ratio (CNR) tended to increase when using high mAs, SNR, and the Clearview IR protocol. Noise decreased when using a high radiation dose and a high percentage of Clearview IR. CTDI_vol_ and DLP were increased with increasing kVp, mAs, and SNR levels, while the increasing percentage of Clearview did not affect the radiation dose. Optimized protocols, including radiation dose and image quality, should be evaluated to preserve diagnostic capability. The recommended parameter settings include kVp set between 100 and 120 kVp, mAs ranging from 200 to 300 mAs, SNR level within the range of 0.7–1.0, and an iterative reconstruction value of 30% Clearview to 60% or higher.

## 1. Introduction

Computed tomography examinations have caused high radiation doses for patients, especially for CT scans of the brain [1]. Currently, CT machines use various technologies or innovations to reduce the radiation dose to patients or to ensure the most negligible radiation dose. In addition, there are also efforts to use low-radiation protocols and develop algorithms or image processing to improve image quality, resulting in better image quality [2,3,4,5,6,7]. Historically, CT protocols were established by the vendor and were not modified or optimized after being used for a long time. Image quality and radiation dose were not considered.

In radiation protection principles, optimization is considered an essential process to ensure that patients receive the appropriate amount of radiation for the examination and that image quality is sufficient for diagnosis [8,9,10,11]. Computed tomography (CT) is a diagnostic imaging modality that uses high radiation doses to create images and may expose the patient to radiation risk [12,13]. CT scans of the head expose sensitive tissue, especially the lenses of the eyes, which can be damaged and develop cataracts if the radiation dose exceeds a certain level [13,14,15,16,17].

According to a report by the United States and Canada, CT scan machines have been widely used in many countries and continued to increase among adults, but at a slower pace in more recent years from 2000 and 2016 [18]. The United Nations Scientific Committee on the Effects of Atomic Radiation (UNSCEAR) reported an average annual effective dose received in the United States. The significant increase in the use of ionizing radiation for medicinal reasons resulted in a total increase from 3.0 mSv in 1980 to 6.2 mSv in 2006. CT scan data showed 1.5 mSv of effective dose and contributed a radiation dose to patients of approximately 24.2% of the normal dose received in daily life [1,18]. Many parameters affect the patient’s radiation exposure, such as kVp, mA, mAs, pitch, scan range, slice thickness, and the reconstruction algorithm. Acquisition parameters also affect image quality and the performance of images for diagnosis [15,19,20,21,22].

Several iterative reconstruction (IR) algorithms are currently available from various CT vendors. IR algorithms are designed to reduce image noise, improve low-contrast detail, reduce artifacts and radiation dose, and maintain high contract resolution. Iterative reconstruction (IR) algorithms are divided into two types: statistical IR algorithms or hybrid and model-based IR algorithms (MBIR) [23]. Statistical IR algorithms are performed using iterative filtration in projection and image space. ASIR, ASIR-V, AIDR3D, iDose, and SAFIRE are commercial statistical IR. MBIR algorithms which are performed using forward projection from the current image estimate in image space to projection space for simulating projection data. After comparing simulated and measured projection data, an image data update can be computed via the back projection of a correction term. The iterative cycle is repeated until a predefined stopping point is reached. VEO, IMR, ADMIRE, and FIRST were the MBIR algorithms [23,24]. ClearView IR operates iteratively in both projection space and image space. The noise that accompanies low-dose acquisitions can be removed while preserving all edges.

Objective and subjective evaluation methods can be used to analyze image quality in CT images [25,26,27,28]. In the objective evaluation method, images are evaluated quantitatively using the QA CT phantom. The subjective evaluation method involved the image quality being evaluated by radiologists or experts, who score the image quality. The typical quantitative parameters used to evaluate the image quality include CT number accuracy and linearity, high-contrast spatial resolution, modulation transfer function (MTF), low-contrast detectability and contrast-to-noise ratio (CNR), image noise, uniformity, and mean CT number [29,30].

Optimization protocols should balance radiation and image quality because acquisition parameter changes could affect the image quality. Therefore, the objectives of this study were to optimize the CT brain protocols by varying kVp, mAs, SNR, and the Clearview iterative reconstruction (IR) algorithm and to evaluate the image quality using the qualitative method, using Catphan700 phantom to obtain the image. Image quality evaluation can use AutoQA Plus software and demonstrate CT number accuracy and linearity, high-contrast spatial resolution, modulation transfer function (MTF), low-contrast detectability and contrast-to-noise ratio (CNR), image noise, uniformity, and mean CT number.

## 2. Materials and Methods

This study used a Catphan700 phantom to acquire images, and the CT scanner was Neusoft in a model of NeuViz 128 (Neusoft Medical Systems, Shenyang, China) with 128 slices. The optimized protocols were adjusted by varying kVp, mAs, SNR, and Clearview iterative reconstruction. After finishing the scanning, a radiation dose of CT was recorded as CTDIvol and DLP.

### 2.1. Image Acquisition Protocols

The scan parameters are presented in Table 1. All CT scans were performed with the same scan parameters, and all data acquisitions were taken 3 consecutive times.

### 2.2. Data Acquisition and Image Quality Evaluation

The Catphan 700 phantom was used to acquire the image by scanning according to three clinical routine scans for the brain. The AutoQA Plus v.1.8.7.0 software (QA Benchmark, LLC, Ellicott City, MD, USA) was used to analyze image quality.

### 2.3. Catphan 700 Phantom

A Catphan 700 phantom (The Phantom Laboratory Incorporated, Salem, NY, USA) was used to evaluate all image quality [31,32]. The phantom has a cylindrical shape and contains 6 modules, including CTP682 (geometry sensitometry and point source module), CTP714 (30-line pair high-resolution module), CTP515 (subslice and supra-slice low contrast), CTP721 (wave insert), CTP723 (bead blocks), and CTP712 (uniformity section). CT scans of the Catphan phantom were obtained using Neusoft NeuViz 128 (Neusoft Medical Systems, Shenyang, China) [33]. Quality control (QC) testing was performed annually for all CT scanners and the CT number was also calibrated.

### 2.4. CT Number Accuracy and Linearity

The module CTP682, containing different sensitometry targets, was used to perform CT number accuracy and linearity [34,35,36]. This module has sensitometry targets made from Teflon^®^, Bone 50%, Delrin^®^, Bone 20%, acrylic, Polystyrene and low-density polyethylene (LDPE), polymethylpentene (PMP), Lung foam #7112, and air, including a water container. In the circular region of interest (ROI), approximately 80% of each target size was selected and the measured mean CT number was recorded for each target. The mean CT number of each target was compared to the range of actual CT numbers from the specifications of the phantom. The linearity was also tested using Pearson’s correlation coefficient (r) between the measured CT number and each target’s linear attenuation coefficients (µ). The CT numbers’ accuracy should not exceed the tolerance limit from the recommendation range of the Catphan 700 phantom.

### 2.5. The High-Contrast Spatial Resolution and Modulation Transfer Function (MTF)

High-contrast spatial resolution is the ability of a system to distinguish high-contrast objects from neighboring objects [37]. Two broad methods exist to analyze high-contrast spatial resolution by calculating the modulation transfer function (MTF) and objective analysis or resolution bar pattern assessment [37,38,39]. The spatial resolution is measured by calculating a small wire’s point spread function (PSF) with 0.05 mm tungsten (module CTP682). PSF generates line spread functions (LSFs) in both vertical and horizontal directions. The MTF was calculated by taking the Fourier transform and shown in the value line pair/cm at 50%, 10%, and 2% of the MTF. The CTP714 high-resolution module with 1–30 line pair per cm gauges was used to evaluate high resolution. The tolerance levels of spatial resolution in the CT brain scan should exceed 5 lp/cm [36]. The expected values of MTF at 50%, 10%, and 2% exceeded 3, 5, and 7 cycles/cm, respectively [31,40,41].

### 2.6. Low-Contrast Detectability and Contrast-to-Noise Ratio (CNR)

Low-contrast resolution refers to the ability of a system to distinguish between low-contrast structures and their background [6,34,42,43]. Module CTP515 was used to determine CNR, which contains low-contrast supra-slice targets with diameters of 15, 9, 8, 7, 6, 5, 4, 3, and 2 mm, and contrast levels of 0.3%, 0.5%, and 1.0%. CNR measured the difference between target signals and background signals. Low-contrast detectability was also calculated and shown as the theoretical contrast–detail curve. The curve showed the minimum contrast level at the given diameter that should be visible. CNR performance should meet the standards at 1 for the adult head protocol [34].

### 2.7. Image Noise, Uniformity, and Mean CT Number

The CTP71 was used for the measurement of uniformity and image noise [31,34,36,44]. Image uniformity was measured by using the difference value of the maximum HU of the center and the 4 peripheral ROI at 3, 6, 9, and 12 o’clock locations. The noise level was defined as SD and measured at the center with a diameter of ROI 40% of the phantom. The mean CT number represents the center ROI mean for a phantom’s 10% ROI size. The difference between the mean CT value of each peripheral ROI and the center ROI should not exceed 5 HU and the noise level should not exceed 5 [34]. The mean CT number should not exceed 12 ± 10 HU.

### 2.8. Radiation Dose

The CTDIvol and dose length product (DLP) were used as dose indices for the CT and collected from the dose report. The DLP was calculated by multiplying the CTDIvol by the scan length [21,45].

### 2.9. Statistical Analysis

Quantitative data from image quality evaluation and radiation dose were expressed as means. CT number linearity was tested by using Pearson’s correlation. CT accuracy was compared with the tolerance values of recommendation. The parameters for the evaluation of image quality were analyzed using AutoQA Plus software and compared with the default protocol or the reference and tolerance values of the American College of Radiology (ACR) or the International Electrotechnical Commission (IEC). Radiation doses (CTDIvol, DLP) were compared with the default protocol and the percentage difference from the default protocol was also calculated. Statistical analysis was performed with one-way ANOVA followed by Tukey post hoc test for comparisons between groups.

## 3. Results

### 3.1. CT Number Accuracy

Table 2 shows that the correlation coefficient found was between 0.998 and 0.999. It indicates that CT numbers and linear attenuation coefficients have a very high positive relationship. For the default clinical brain protocols at 120 kVp, 300 mAs, SNR 1.0, and 50% Clearview IR algorithm, the CT numbers did not pass the evaluation criteria for Teflon and Delrin. When the kVp was changed to 80 kVp, Acrylic, Bone 50%, LDPE, Bone 20%, Polystyrene, and PMP did not pass the criteria, while Teflon and Delrin did not pass the criteria at 100 kVp. At 140 kVp, Bone 20% and Delrin did not pass the criteria. When the mAs were changed to 100, 200, and 400 mAs, with SNR levels of 0.3, 0.7, 1.3, and 1.7, and 20%, 30%, 40%, and 60% Clearview IR, Teflon and Delrin did not pass the criteria.

### 3.2. Modulation Transfer Function (MTF)

All optimized protocols demonstrated that the MTF passed the evaluation criteria for all percentages of MTF at 50%, 10%, and 2%, with tolerance levels of 3, 5, and 7 cycles/cm, respectively (Table 3). Varying kVp revealed that protocols at 100 and 120 kVp had an MTF value that was 50%, 10%, and 2% higher than protocols at 80 and 140 kVp (*p* < 0.001). When the mAs were optimized to 100, 200, 300, and 400 mAs, it was found that the MTF values at 50%, 10%, and 2% showed a higher MTF when the mAs were increased. When the SNR levels were varied to 0.3, 0.7, 1.0, 1.3, and 1.7, it was found that the MTF value increased as the SNR level increased. Finally, when the IR was at 20%, 30%, 40%, 50%, and 60% Clearview, it was discovered that the MTF value showed a similar value of MTF at 30%, 40%, 50%, and 60% Clearview, while a 20% MTF showed the worst MTF value.

### 3.3. High-Contrast Spatial Resolution

**Figure 1:**
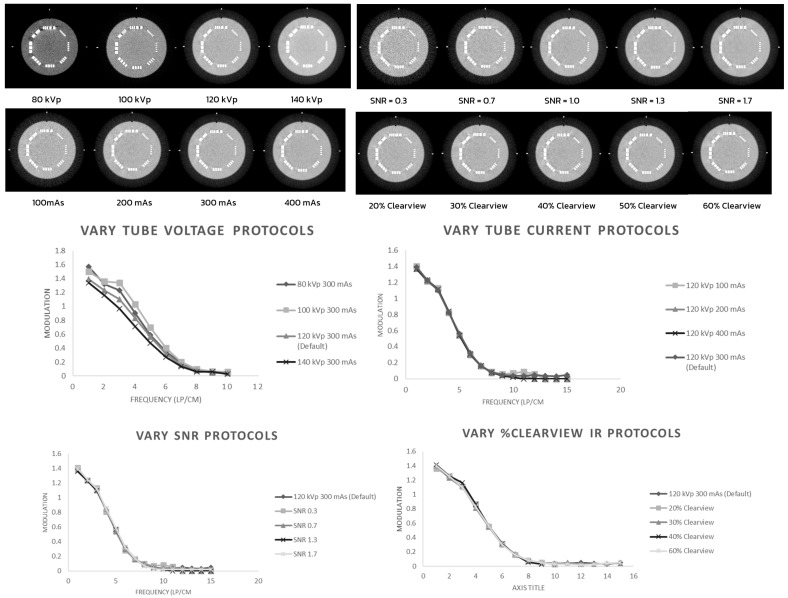
High-contrast spatial resolution of optimized protocols.

Figure 1 shows that the 80 kVp and 100 kVp protocols had higher high-contrast spatial resolution than the 120 kVp and 140 kVp protocols (*p* < 0.001). The mAs, SNR, and percentage Clearview IR algorithm did not affect the high-contrast spatial resolution.

### 3.4. Low-Contrast Detectability

**Figure 2:**
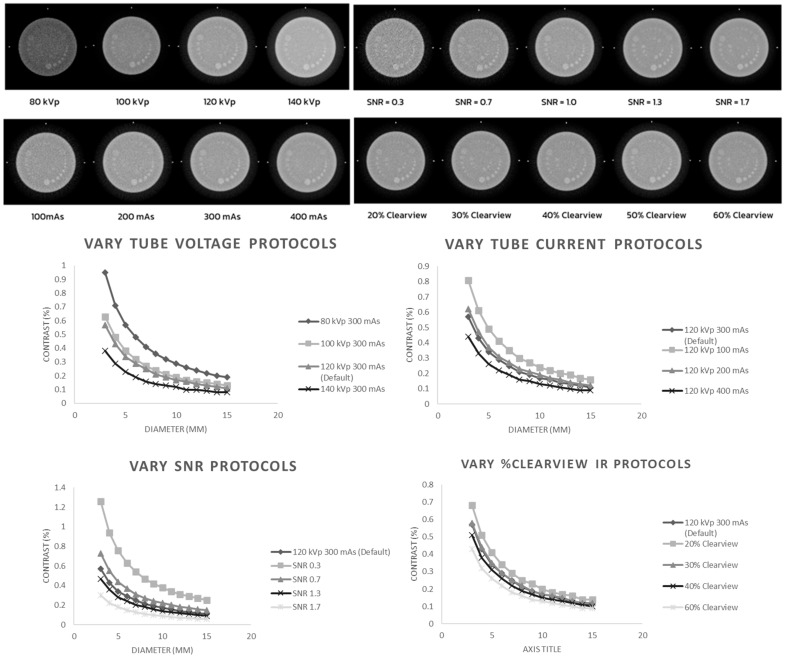
Low-contrast detectability of optimized protocols.

Figure 2 shows low-contrast detectability in the C-D model. It was found that increasing the kVp, mAs, SNR, and % Clearview IR caused an improvement in the low-contrast detectability in the low-contrast object and for objects with a small diameter. Conversely, decreasing the kVp, mAs, SNR, and % Clearview IR caused a decrease in low-contrast detectability.

### 3.5. Contrast-to-Noise Ratio (CNR)

**Figure 3:**
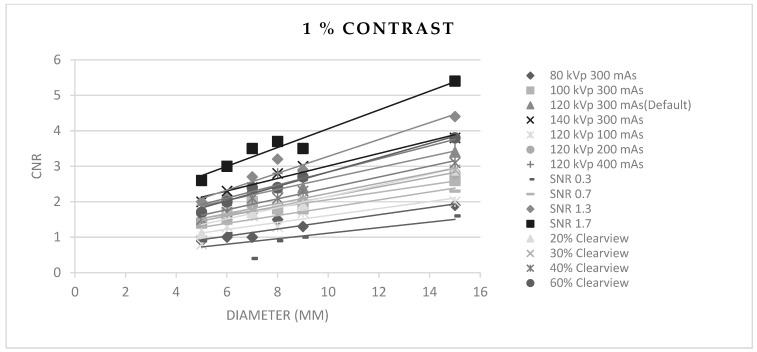
Contrast-to-noise ratio at 1% contrast with various diameters of optimized protocols.

**Figure 4:**
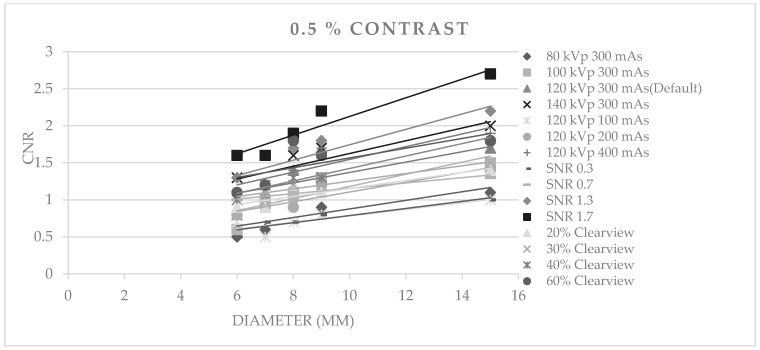
Contrast-to-noise ratio at 0.5% contrast with various diameters of optimized protocols.

**Figure 5:**
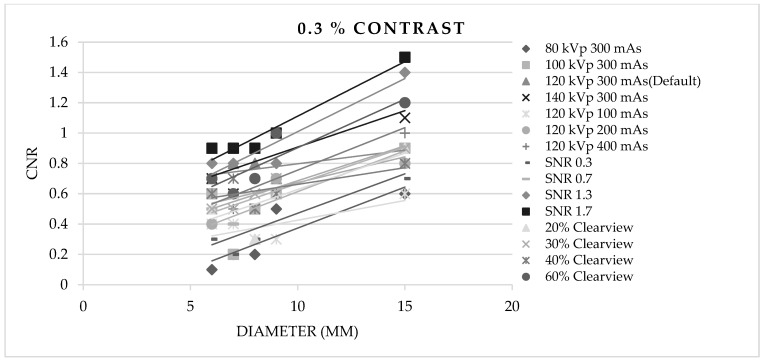
Contrast-to-noise ratio at 0.3% contrast with various diameters of optimized protocols.

Figure 3, Figure 4 and Figure 5 show CNR at percentage contrast objects of 1%, 0.5%, and 0.3% with various object diameters. It was found that increasing the radiation dose by increasing kVp, mAs, and SNR produced a higher CNR, especially for protocols of SNR 1.7 and SNR 1.3. Increasing the % Clearview IR also caused a higher CNR. Conversely, decreasing radiation via decreased kVp, mAs, and SNR showed a decrease in low-contrast detectability. The higher % Clearview IR showed an improvement in CNR.

### 3.6. Image Noise, Uniformity, and Mean CT Number

**Figure 6:**
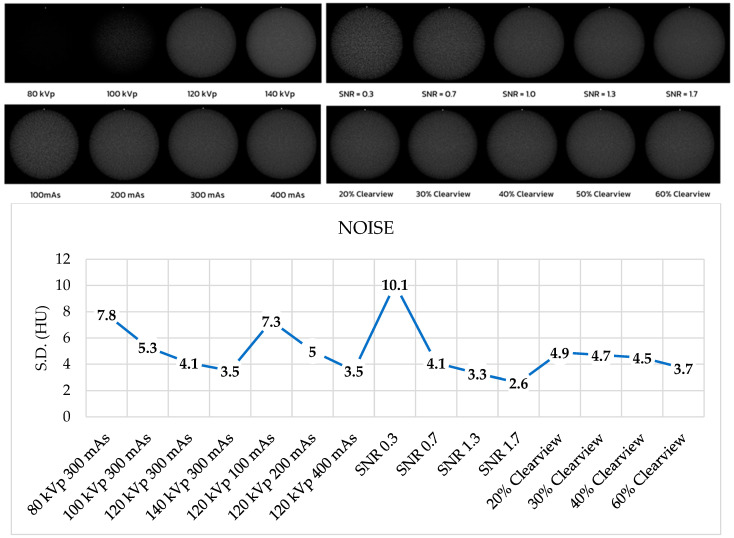
Noise level of optimized protocols.

**Figure 7:**
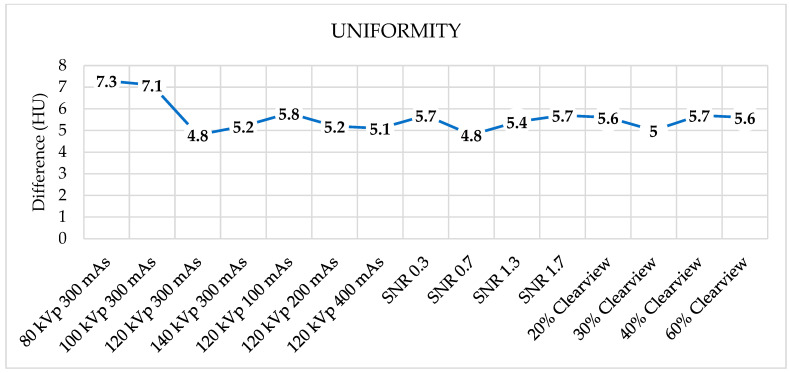
Uniformity of optimized protocols.

**Figure 8:**
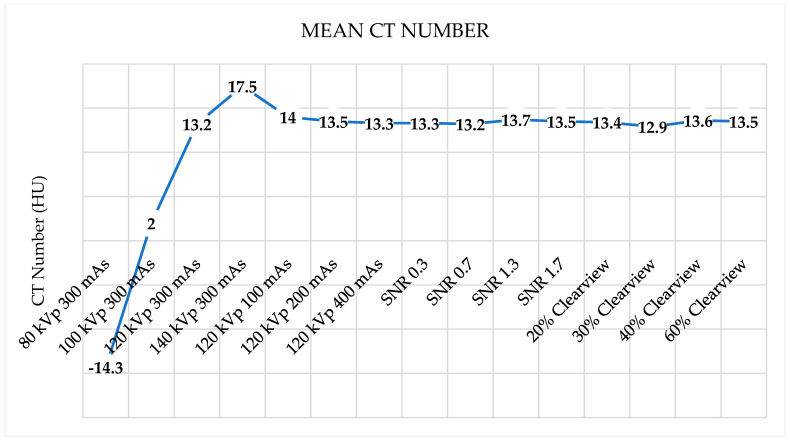
Mean CT number of optimized protocols.

Figure 6 shows the noise levels from various optimized protocols. Noise levels were found to be acceptable within a tolerance of 5 HU for protocols of 120 kVp, 140 kVp, 200 mAs, 400 mAs, SNR 0.7, SNR 1.3, SNR 1.7, and all percentages of the Clearview IR algorithm. Figure 7 shows that all protocols failed to meet the acceptable range of 4 HU. Figure 8 demonstrates that the mean CT number of all protocols was within the acceptable range of 12 ± 10 HU, except for protocol 80 kVp, which had a low mean CT number.

### 3.7. Radiation Dose

**Figure 9:**
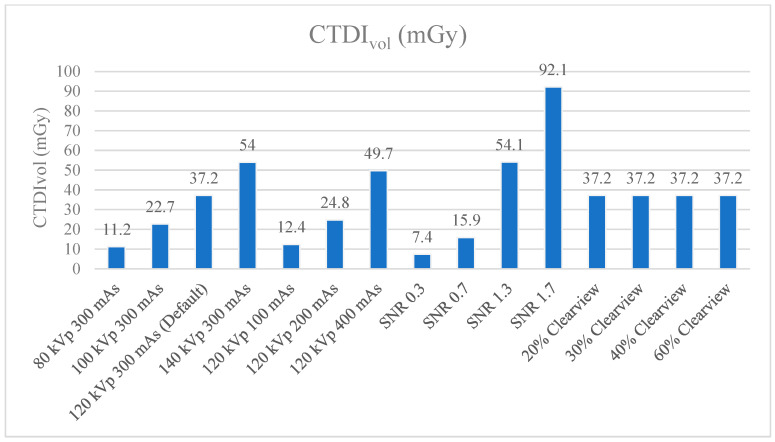
CTDI_vol_ (mGy) of optimized protocols.

**Figure 10:**
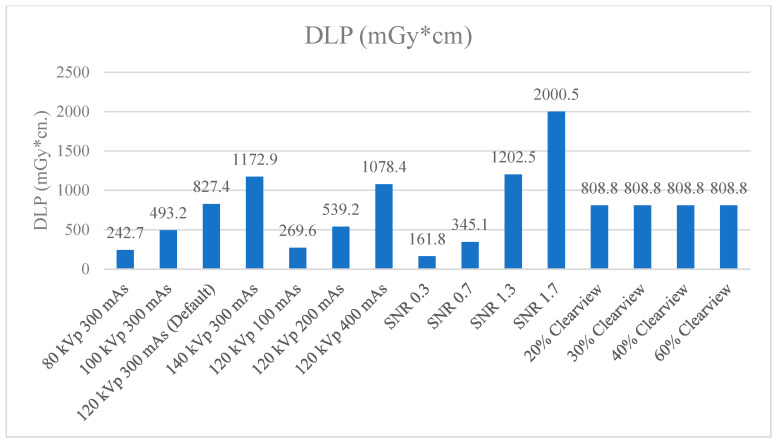
DLP (mGy*cm) of optimized protocols.

Figure 9 and Figure 10 show the CTDIvol and DLP of the optimized protocols. The default clinical protocol of brain CT exhibited CTDIvol and DPL at 37.2 mGy and 827.4 mGy*cm, respectively. It was found that protocols of 80 kVp, 100 kVp, 100 mAs, 200 mAs, SNR 0.3, and SNR 0.7 had lower CTDIvol and DPL than the default clinical protocol (*p* < 0.001), while 140 kVp, 400 mAs, SNR 1.3, and SNR 1.7 showed higher CTDIvol and DLP than the default clinical protocol (*p* < 0.001). CTDIvol and DLP were not affected when the levels of the Clearview IR algorithm changed. Table 4 shows the percentage difference in CTDIvol and DLP compared to the default clinical protocol. The lower CTDIvol and DPL protocol could decrease CTDIvol to 33.3–80.1% mGy and DLP to 33.4–80.4% mGy*cm. A higher dose protocol could increase CTDIvol to 33.6–147.6% mGy and DLP to 30.3–141.8% mGy*cm.

## 4. Discussion

Optimization is considered an essential process in the principles of radiation protection to ensure that patients receive the appropriate amount of radiation for the examination and that image quality is sufficient for diagnosis. Computed tomography (CT) is one of the diagnostic imaging modalities that uses high radiation doses to form images and may contribute to the radiation risk to the patient. CT scans of the head expose sensitive organs, particularly the lenses of the eyes, which may be damaged and develop cataracts if the radiation dose exceeds a certain threshold. This study aimed to optimize the protocol for CT brain scans, a routine protocol used in clinical practice. The protocol was adjusted with the following scanning parameters: kVp (80, 100, 120, 140), mAs (100, 200, 300, 400), SNR (0.3, 0.7, 1, 1.3, 1.7), and the Iterative Reconstruction algorithm (20%, 30%, 40%, 50%, 60% Clearview). Moreover, quantitative image quality was also evaluated using the Catphan700 phantom to acquire images and evaluate the CT number accuracy and linearity, MTF, high-contrast spatial resolution, low-contrast detectability, CNR, image noise and uniformity, and mean CT number. CTDIvol and DLP values were also recorded and evaluated in various adjusted brain protocols.

The results demonstrated that CT number accuracy was within the specified range for most materials and showed a linear relationship between each material’s linear absorption coefficient (µ) and CT number. The correlation coefficient (*r*) was within the range of 0.998165 to 0.999701, showing a strong positive relationship. For the 80 kVp protocol adjustment, it was found that the CT number accuracy showed out-of-range CT numbers in many materials because the average energy was decreasing, affecting the absorption coefficient (µ) and resulting in the CT number increasing [35]. Usually, 120 kVp is used for CT scans; this machine has an effective energy of approximately 60 keV. Reducing the energy to 80 kVp will affect the average energy and CT number accuracy. In addition, the factors affecting the CT number accuracy depend on the filter and patient’s slice thickness [46,47]. Therefore, the CT number measurement for diagnosis should be considered carefully because of the high error of CT number six. Some materials in another protocol also showed an error in CT number accuracy. It has been recommended that water calibration should be performed regularly to help maintain the accuracy of CT numbers, especially the CT number accuracy of the water material [36].

High-contrast spatial resolution is used to distinguish small objects with high contrast. It can be measured in two ways, using MTF and high-resolution bar patterns. MTF values of 50%, 10%, and 2% of all protocols were found to have passed the 3 lp/cm, 5 lp/cm, and 7 lp/cm benchmarks, respectively. The results demonstrated that the 80 and 140 kVp protocol showed lower MTF values than 100 and 120 kVp because of the increasing noise at 80 kVp and the scatter radiation at 140 kVp [48]. Increasing mAS and SNR could increase radiation, resulting in low noise levels. The 20% Clearview IR showed the worst MTF because the power of IR at 20% was not enough to improve the image quality. High-resolution assessments using bar patterns at 80 and 100 kVp protocols showed higher high-contrast spatial resolution because the lower kVp could increase the high-contrast resolution of the images. According to the previous study, the factors affecting MTF and spatial resolution were not only the reconstruction algorithms, but also the detector width, effective slice thickness, object-to-detector distance, X-ray tube focal spot size, and matrix size. These factors also affect MTF and spatial resolution [49,50]. Research by Yali Li et al. found that 10% of MTF was 6.98 ± 0.40 lp/cm, correlating with a subjective assessment showing 7 lp/cm. Moreover, Clearview is suitable for low-dose protocols because it reduces noise and artifacts [42]

Low-contrast detectability is the ability to separate low-contrast objects from the background. It was found that protocols that use low radiation doses tend to have decreased low-contrast resolution, such as the SNR 0.3 protocol, 80 kVp protocol, and 100 kVp protocol. The protocols with improved low-contrast resolution were the SNR 1.7 protocol, 140 kVp protocol, and 60% Clearview protocol. In addition, it was found that the CNR value decreased with the percentage contrast of the object decrease and the small diameter of the object. Studies by Manson EN et al. and Gulliksrud K et al. showed that noise was an essential factor that reduced low-contrast resolution and CNR [51,52]. Moreover, higher radiation dose protocols and a higher percentage of reconstruction algorithms could improve the low-contrast resolution and CNR [51].

The evaluation of image uniformity was undertaken to measure the stability of the CT number by comparing the CT number values in the center of the phantom with the peripheral edges of the phantom. It was found that the uniformity of the images was not within the criteria specified in every protocol, with values exceeding ±4 HU. Noise is the average of the standard deviation (SD) measured at the center of the phantom. Protocols that use high radiation doses, such as high mAs, high kVp, and high IR % Clearview, tend to decrease noise. Research by Yali Li et al. found that the protocol that increased the radiation dose and iterative reconstruction algorithm reduced noise and improved the image quality [42].

CTDIvol and DLP were used to evaluate the radiation dose and to act as a dose index in the CT. Annual quality controls are conducted in CT to measure the CTDIvol, and the value shown in the CT display was not different from the acceptable threshold of ±20%. The DLP value obtained from CTDIvol multiplied by scan length according to the clinical default protocol of CT brain showed 37.2 mGy of CTDIvol. Increasing mAs, kVp, and SNR showed a higher CTDIvol, while increasing the percentage of the Clearview IR algorithm did not affect CTDIvol, but did affect image quality [43,53]. Research by Ozdil Baskan et al. [2] demonstrated that many parameters reduced the radiation dose to some extent, such as kVp, mAs, automated tube current modulation, adaptive dose collimation, and appropriate noise-reduction reconstruction algorithms. Adjusting such parameters will affect the image quality; therefore, adjusting the parameters should be considered. The data demonstrate that a higher % Clearview IR could enhance the MTF, CNR, and low-contrast detectability, and suppress the noise level. In other words, when decreasing the radiation dose or using low protocol, IR can improve and maintain image quality in the acceptable range [54,55].

Because this study was carried out on a Catphan phantom, CT images of patients should also be analyzed. Furthermore, optimization was performed in one CT machine from a specific manufacturer; the optimization parameter, algorithm, and image quality result could differ if another manufacturer were used. We propose that this research be conducted in the CT brain’s low-dose protocol to evaluate the performance of the Clearview IR algorithm for noise reduction. Other parameters that affect radiation dose and image quality could be investigated, and radiologists’ subjective evaluations should be included for the overall image quality evaluation. Moreover, the proper training of radiologist and technologist staff is very important to select the most appropriate CT image acquisition and reconstruction parameters to improve image quality and optimize patient radiation exposure. It was found that staff training on radiation dose and dose optimization applied in pediatric CT scans showed efficacy for dose reduction and increasing dose awareness in patients [56,57].

## 5. Conclusions

The default clinical brain protocol should be optimized for image quality and radiation dose balance. kVp should be set between 100 and 120 kVp because adjusting the kVp below 100 kVp results in a large discrepancy in the CT number. The mAs should be in the range of 200–300 mAs, because adjusting the mAs value to 200 will reduce the radiation dose, but the image quality in CNR and low-contrast detectability will be similar to the default brain protocol. The SNR level should be in the range of 0.7–1.0, because adjusting the SNR level to 0.7 will reduce the radiation dose but result in a CNR, low-contrast detectability, and noise within an acceptable range. However, the value of uniformity will increase. Iterative reconstruction should be adjusted to a value between 30% Clearview and 60% Clearview or more. Adjusting Clearview to 30% or more will result in CNR, low-contrast detectability, and noise reduction without affecting radiation dose. However, the noise and non-uniformity of CT images increased when using low-dose protocols.

## Figures and Tables

**Table 1:** Data acquisition protocols used for dose optimization in default clinical brain protocols of the Neusoft NeuViz 128 CT scanner.

Parameters	Default (Optimized Protocol)
kVp	120 (80, 100, 140)
mAs	300 (100, 200, 400)
Rotation time	1.0 s
Pitch	0.5
Slice thickness	5 mm
SNR level	1.0 (0.3, 0.7, 1.3, 1.7)
FOV	250 mm
Kernel	F20
IR	50% (20%, 30%, 40%, 60%) Clearview
Matrix	512 × 12

**Table 2:** CT number accuracy in each optimized protocol and material, and correlation coefficient (r) showing the relationship between CT number and attenuation coefficient.

Materials	80 kVp 300 mAs	100 kVp 300 mAs	120 kVp 300 mAs (Default)	140 kVp 300 mAs	120 kVp 100 mAs	120 kVp 200 mAs	120 kVp 400 mAs	
Air	−973.8	−971.9	−967.8	−968	−968	−968	−969.1	
Lung	−806.5	−805.1	−798.5	−800.3	−798.4	−800	−800	
PMP	−211.2	−190.8	−177.1	−171.1	−176.5	−176.6	−176.5	
LDPE	−123.9	−103.7	−90	−82.1	−89.1	−88.8	−89	
Polystyrene	−67.5	−46.9	−33.6	−27.7	−34.7	−33.3	−33.8	
Water	−0.9	1.3	2.8	0.7	3.5	3.5	3.3	
Acrylic	98.5	112.6	122.6	127.1	123.5	124.6	123.3	
Bone20	302	168.5	186.9	223.6	186.2	187.1	187.5	
Delrin	330.4	244.7	301.1	360.9	300.3	301.3	301.1	
Bone50	908.3	643.1	629.9	636	628.2	631	629	
Teflon	969	813.5	882	931.1	882.4	882.1	883.1	
*r*	0.998	0.998	0.999	0.999	0.999	0.999	0.999	
Materials	SNR 0.3	SNR 0.7	SNR 1.3	SNR 1.7	20% Clearview	30% Clearview	40% Clearview	60% Clearview
Air	−969.6	−968.6	−966.7	−967.6	−969.9	−969.6	−970.2	−965.3
Lung	−800.5	−799.4	−798.3	−797.8	−800.7	−799.7	−801.5	−797.8
PMP	−177.5	−176.9	−177.4	−176.5	−176.6	−176.9	−177.5	−175.9
LDPE	−90.8	−89.3	−88.8	−89.2	−89.3	−89.9	−89.7	−89.6
Polystyrene	−35.5	−34.7	−33.9	−34.3	−34.6	−34.5	−35	−33.6
Water	2.2	2.5	1.8	2.6	2.7	3.7	1.4	3.2
Acrylic	122.6	124.1	123.3	123.8	123.6	123.2	123	123.3
Bone20	188.8	186.4	187.7	188.3	188.8	189.1	187.8	184
Delrin	300.4	299.8	300.1	300	299.3	300.7	299.7	300.8
Bone50	628.6	629.3	628.1	629.1	628.6	628.8	628.4	628.3
Teflon	881.6	881.7	879.7	880.4	882.4	881.1	879.9	882.7
*r*	0.999	0.999	0.999	0.999	0.999	0.999	0.999	0.999

**Table 3:** MTF at 50%, 10%, and 2% of optimized protocols.

MTF (%)	80 kVp 300 mAs	100 kVp 300 mAs	120 kVp 300 mAs (Default)	140 kVp 300 mAs	120 kVp 100 mAs	120 kVp 200 mAs	120 kVp 400 mAs	
50	3.74	4.37	4.18	3.86	4.03	4.28	4.5	
10	6.85	7.07	7.1	6.69	6.97	6.97	6.97	
2	8.33	8.34	8.82	8.41	8.09	8.09	8.31	
MTF (%)	SNR 0.3	SNR 0.7	SNR 1.3	SNR 1.7	20% Clearview	30% Clearview	40% Clearview	60% Clearview
50	4.41	4.41	4.41	4.41	3.95	4.12	4.12	4.29
10	6.87	6.93	6.94	7.02	6.83	6.95	7.12	7.12
2	8.45	8.5	8.52	8.62	8.54	8.55	8.59	8.59

**Table 4:** Percentage difference in CTDIvol and DLP compared to default clinical protocols.

	80 kVp 300 mAs	100 kVp 300 mAs	120 kVp 300 mAs (Default)	140 kVp 300 mAs	120 kVp 100 mAs	120 kVp 200 mAs	120 kVp 400 mAs	
% Diff of CTDIvol	−69.9	−39.0	0.0	45.2	−66.7	−33.3	33.6	
% Diff of DLP	−70.7	−40.4	0.0	41.8	−67.4	−34.8	30.3	
	SNR 0.3	SNR 0.7	SNR 1.3	SNR 1.7	20% Clearview	30% Clearview	40% Clearview	60% Clearview
% Diff of CTDIvol	−80.1	−57.3	45.4	147.6	0.0	0.0	0.0	0.0
% Diff of DLP	−80.4	−58.3	45.3	141.8	0.0	0.0	0.0	0.0

## Data Availability

Data are contained within the article.

## Abbreviations

CNR, contrast-to-noise ratio; CT, computed tomography; HU, Hounsfield units; keV, kiloelectronvolt; kVp, kilovolt peak; DLP, dose length product; SNR, signal-to-noise ratio; IR, iterative reconstruction; MTF, modulation transfer function.
